# Cellular Prion Protein Combined with Galectin-3 and -6 Affects the Infectivity Titer of an Endogenous Retrovirus Assayed in Hippocampal Neuronal Cells

**DOI:** 10.1371/journal.pone.0167293

**Published:** 2016-12-09

**Authors:** Boe-Hyun Kim, Hae-Young Shin, Joy J. Goto, Richard I. Carp, Eun-Kyoung Choi, Yong-Sun Kim

**Affiliations:** 1 Department of Medicine, Division of Infectious Diseases, Icahn School of Medicine at Mount Sinai, New York, New York, United States of America; 2 Ilsong Institute of Life Science, Hallym University, Gwanyang-dong, Dongan-gu, Anyang, Gyeonggi-do, Republic of Korea; 3 Department of Chemistry, California State University, Fresno, California, United States of America; 4 New York State Institute for Basic Research in Developmental Disabilities, Staten Island, New York, United States of America; University of Maryland School of Medicine, UNITED STATES

## Abstract

Prion diseases are infectious and fatal neurodegenerative diseases which require the cellular prion protein, PrP^C^, for development of diseases. The current study shows that the PrP^C^ augments infectivity and plaque formation of a mouse endogenous retrovirus, MuLV. We have established four neuronal cell lines expressing mouse PrP^C^, PrP^+/+^; two express wild type PrP^C^ (MoPrP^*wild*^) and the other two express mutant PrP^C^ (MoPrP^*mut*^). Infection of neuronal cells from various PrP^+/+^ and PrP^-/-^ (MoPrP^*KO*^) lines with MuLV yielded at least three times as many plaques in PrP^+/+^ than in PrP^-/-^. Furthermore, among the four PrP^+/+^ lines, one mutant line, P101L, had at least 2.5 times as many plaques as the other three PrP^+/+^ lines. Plaques in P101L were four times larger than those in other PrP^+/+^ lines. Colocalization of PrP and CAgag was seen in MuLV-infected PrP^+/+^ cells. In the PrP-MuLV interaction, the involvement of galectin-3 and -6 was observed by immunoprecipitation with antibody to PrP^C^. These results suggest that PrP^C^ combined with galectin-3 and -6 can act as a receptor for MuLV. P101L, the disease form of mutant PrP^C^ results suggest the genetic mutant form of PrP^C^ may be more susceptible to viral infection.

## Introduction

Transmissible spongiform encephalopathies (TSEs), or prion diseases, are infectious and fatal neurodegenerative diseases occurring in humans and animals. These diseases include Gerstmann-Sträussler-Scheinker (GSS) syndrome, Familial Fatal Insomnia (FFI), Creutzfeldt-Jakob disease (CJD) and kuru in humans, and a group of animal diseases that includes scrapie in sheep and goats, bovine spongiform encephalopathy (BSE) in cattle, and chronic wasting disease in mule deer and elk [1, 2]. In prion disease, the cellular form of the prion protein, PrP^C^, is absolutely needed for developing the disease; PrP^C^-deficient mice are not susceptible to prion disease [3]. The conformational conversion of PrP^C^ into abnormal protease-resistant PrP^Sc^ is important event in the pathogenesis of TSEs [4, 5], and PrP^Sc^ or PrP^Sc^-like peptides have been reported to inhibit the function of PrP^C^ and affect the function of copper homeostasis, leukoctye differentiation or neurogenesis [6]. PrP^C^ is mainly localized in cellular membranes by glycosyl-phosphatidylinositol (GPI) anchor [7]. There are numerous proteins that have been proposed to interact with PrP^C^; the anti-apoptotic molecule Bcl-2 [8, 9], the chaperone Hsp60 [10], the laminin receptor precursor LRP [11], synapsin, the adaptor protein Grb2 and the protein Pint 1 (prion interactor 1) [12]. PrP^C^ is also known as a synaptic vesicle-forming protein [12], copper-binding protein [13, 14], and as an antioxidant during oxidative stress [15, 16]. The identity of the function of PrP^C^ in the pathogenesis of prion disease needs to be clarified.

Galectins belong to a family of proteins, which contain carbohydrate recognition domains with affinity for β-galactosides [17, 18]. Galectin proteins have a role in the recognition of endogenous carbohydrate ligands in embryogenesis, development and immune regulation [19]. Galectin proteins are subdivided into three types, the proto type, the chimera type, and the tandem repeat type, and so far, 15 members have been described in mammals [20]. Recently, galectins have been discovered to bind glycans on the surface of viruses and other microorganisms. Of the galectin members, galectin-1 and galectin-3 are the most extensively studied and are known as important mediators of inflammation; galectin-1 belongs to the prototype galectins and galectin-3 is a chimeric galectin [19]. Galectin-1 has been documented in virus recognition and host cell fusion by binding the virion envelope or capsid glycoproteins then promoting cell-cell fusion and formation of syncytia [21, 22]. Galectin-3 has been shown to play a pivotal role in diverse physiological functions and in pathological processes as an inflammatory mediator [21, 23].

We investigated whether any change occurs in the expression of galectins as a result of murine endogenous retrovirus (MuLV) infection in hippocampal neuronal cells. Previously, we have reported the change of galectin-3 protein expression in murine prion disease model, scrapie, and showed its correlation with PrP^Sc^ accumulation in scrapie [24]. Therefore, we examined the possible association of galectins and PrP^C^ in virus infection. In this study, we used wild-type, octarepeat deletion type, and P101L mutant form of PrP^C^ which is equivalent to the GSS mutation, one of the human prion diseases. We showed that PrP^C^ has a role as a viral receptor for MuLV in neuronal cells. The evidence for PrP^C^ as a receptor derives from MuLV plaque titer and plaque size in PrP^+/+^ compared to PrP^-/-^ cells. PrP^C^ also appears to regulate the expression of several galectins.

## Results

### Establishment of *Prnp*^-/-^ astroglia cell lines from PrP^C^-deficient Zürich I PrP^-/-^ (Zür I) mice and selection of stable neuronal cells

**Fig 1:**
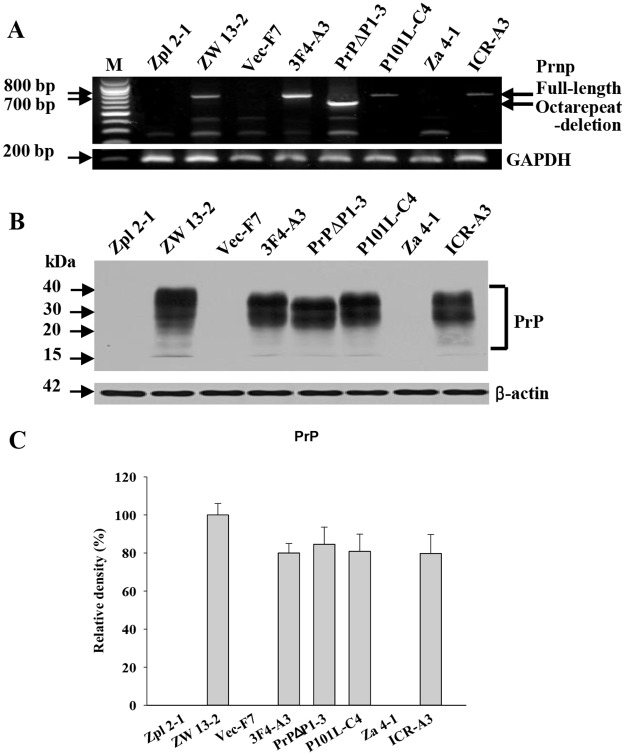
Expression levels of the PrP^C^ genes and proteins in neuronal and astroglial cell lines. (A) Analysis of expression levels of *Prnp* in *Prnp*^-/-^, Zpl, Vec, and Za, and *Prnp*^+/+^, ZW, 3F4, PrPΔ, P101L, and ICR cell lines. ZW, 3F4, P101L, and ICR-A cell lines contained full-length of *Prnp* (789 bp). Cell lines expressing wild-type PrP^C^ are called the ZW cell line (Table 1). PrPΔ cell line contained shorter length *Prnp* (663 bp). Zpl, Vec, and Za cell lines were negative for *Prnp* detection. (B) Protein levels of PrP in cell lines were consistent with the results of RT-PCR analysis. PrPΔ cell line showed shorter length PrP. (C) Densitometry analysis of PrP protein expression showed no significant difference between wild-type cells and PrP-transfected cells. Relative values are represented as the mean±SEM. Cell lines were assessed by three separate experiments. ZW 13–2, 100±7.82; 3F4-A3, 87.8±9.53; PrPΔP1-3, 93.8±9.11; P101L-C4, 83.94±9.41; ICR-A3, 88.75±10.23.

Neuronal cell lines expressing wild-type PrP^C^ and PrP^C^-deficient, named ZW and Zpl cell lines, were previously established (Table 1)[25]. PrP^-/-^ neuronal cells, Zpl 2–1, 2–4, 3–4, were transfected with the following DNA constructs: wild-type PrP^C^ DNA containing the 3F4 epitope (3F4), or wild-type DNA containing deletion of the octapeptide repeat (PrP(Δ53–94)), or wild-type DNA with GSS mutation (P101L). Additional lines were transfected with vector alone (Fig 1, S1 Fig). We selected three clonal lines that expressed wild-type PrP^C^ with 3F4 epitope (3F4-A3, 3F4-A6, and 3F4-C5), three lines that expressed octarepeat deletion-type PrP^C^ (PrPΔH3-2, PrPΔP1-3, PrPΔP3-2), and three lines that expressed P101L-type PrP^C^ (P101L-C4, P101L-E6, P101L-E9). Empty vector transfected clones were also selected (Vec-F5, Vec-F6, Vec-F7). Expression of PrP^C^ was evaluated by RT-PCR and Western blot; one clone from each line is shown in Fig 1, other clones from each line gave similar results. GAPDH (glyceraldehyde-3-phosphate dehydrogenase) and β-actin were used as the housekeeping control for gene and protein expression, respectively.

**Table 1:** Origin and derivation of cell lines.

Designation	Cell type	Derivation
Zpl	Neuronal	Zürich I, PrP^C^ knockout mouse
ZW	Neuronal	ICR, wild type mouse origin, control for Zpl cell line
Vec	Neuronal	Control for transfected Zpl cell lines with vector
3F4	Neuronal	Transfected Zpl cell lines with mouse PrP^C^ containing 3F4 epitope
PrPΔ	Neuronal	Transfected Zpl cell lines with PrP^C^ lacking octapeptide repeat
P101L	Neuronal	Transfected Zpl cell lines with PrP^C^ containing GSS mutation site
Za	Astroglial	Glial cell line from Zürich I PrP^C^ knockout mouse
ICR-A	Astroglial	PrP^C^-positive glial cell line from ICR wild type mouse

Three distinct astroglial cell lines were established from the cortex of Zür I mice (Za 4–1, 4–2, 4–3); as controls, three astroglial cell lines expressing wild-type PrP^C^ (ICR-A1, -A2, -A3) were established from ICR mice (PrP^+/+^) [26]. The gene SV40 large T antigen (SV40LT-Ag) was used to immortalize cells and was then detected as an immortalization marker by Western blot analysis (S1 Fig). PrP-deficient and PrP^C^ expression in established cells were detected by Western blot analysis (S1 Fig). β-actin was used as the housekeeping control protein. The cell-type marker antibodies, anti-GFAP (glial fibrillary acidic protein) for astroglia cells, anti-MAP2 (microtubule-associated protein 2) for neuronal cells, and anti-CNPase (2',3'-cyclic nucleotide 3'-phosphodiesterase) for oligodendrocyte cells were used to determine cell type. We selected Za cell lines which were only positive for astroglia marker in both Western blot and immunofluorescence analysis (S1 Fig).

### Neuronal cells expressing PrP^C^ are more susceptible to MuLV infection than cells without PrP^C^

**Fig 2:**
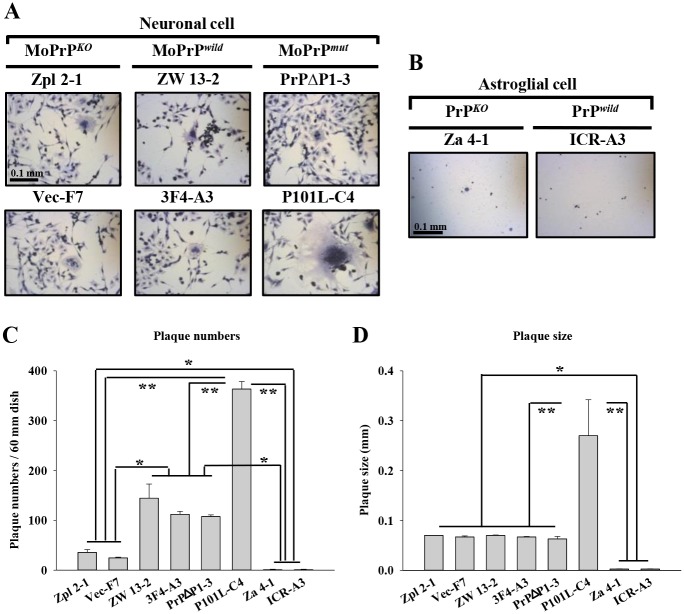
Association of PrP^C^ and morphological appearance of plaques in PrP^-/-^ and PrP^+/+^ neuronal and astroglial cells. The XC/UV plaque assay was applied to each of the cell lines listed in S1 and S2 Tables. (A) The average plaques numbers of each cell lines were 35.6±5.68 from Zpl, 144.3±28.43 from ZW, 24.6±1.52 from Vec, 112±6.08 from 3F4, 107±3.21 from PrPΔ, 363±15.17 from P101L, and 1.5±1 from each of Za and ICR (S1 Table). The shape and size of the plaques were similar in the 2 *Prnp*^-/-^ lines (MoPrP^*KO*^) and in ZW, 3F4 (MoPrP^*wild*^) and PrPΔ cell lines (MoPrP^*mut*^). In P101L cells (MoPrP^*mut*^), the plaques were approximately 4 times larger (S2 Table), and the shape was more round and floral than seen in the other lines. (B) Astroglial cells did not form plaques like neuronal cells. The number of plaques was very low, and the average size was 25-fold smaller than those seen in neuronal cells (S1 Table). MoPrP^*KO*^ or MoPrP^*wild*^ did not affect plaque formation in astroglial cells. Scale bar = 0.1 mm. (C-D) Quantity of plaque numbers and size were measured. There is a significant difference in plaque numbers and size of P101L (MoPrP^*mut*^) (**p < 0.001) and *Prnp*^-/-^ lines (MoPrP^*KO*^) and in ZW, 3F4 (MoPrP^*wild*^) and PrPΔ cell lines (MoPrP^*mut*^)(*p < 0.01).

We infected MuLV to neuronal and astroglial cell lines with or without PrP to assay correlation of PrP and MuLV infectivity Fig 2, S2 Fig. MuLV was extracted from 12-month-old SAMP8 mouse brains then titred by XC/UV plaque assay. We found that neuronal cells with and without PrP^C^ could be infected with MuLV, however, astroglial cells were resistant regardless of their PrP^C^ status (Fig 2, S2 Fig). Although both PrP^-/-^ and PrP^+/+^ neuronal cells were infectable with MuLV, their infection rates were quite different (S1 Table). PrP^C^-deficient cell lines, Zpl and Vec, were showed 35.6 ± 5.68 and 24.6 ± 1.52 plaques. ZW and 3F4 cell lines which were expressing normal wild-type PrP^C^ showed 144.3 ± 28.43 and 112 ± 6.08 plaques. One of the PrP^C^ mutant cell lines which were expressing octarepeat deletion PrP, (PrPΔ) showed 107±3.21 numbers of plaques and the other mutant P101L which were equivalent to human GSS syndrome formed 363 ± 15.17 plaques. MuLV was only able to form plaques in neuronal cells not in astroglial cells. Compared with PrP^-/-^ neuronal cells, wild-type and two of the PrP^C^ transfected lines were approximately 3-fold more susceptible to MuLV infection (*p* < 0.001). Another interesting finding was the fact that neuronal cells containing PrP with the GSS mutation (P101L) showed 2.5-fold higher MuLV plaque numbers compared with the other three PrP^+/+^ cell lines (*p* < 0.001). The infectivity was measured by counting plaque numbers using the UV plaque assay (S1 Table).

The plaque size was measured after infection with MuLV (S2 Table). Five neuronal cell lines, Zpl, ZW, Vec, 3F4, and PrPΔ53–94, had a plaque size in the range of 0.05 mm– 0.09 mm. In contrast, P101L cells had a plaque size (0.18–0.37 mm), approximately four times larger than in the other neuronal cells. Astroglial cells, Za and ICR-A, had a plaque size in the range of 0.002 mm– 0.003 mm.

### Localization of PrP and the effect of MuLV infection on PrP

**Fig 3:**
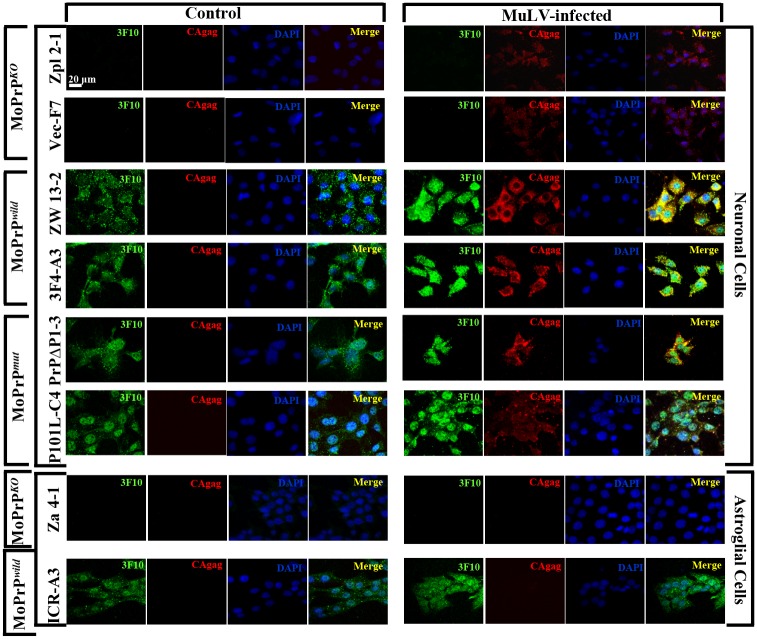
Different susceptibility to MuLV infection of MoPrP^*KO*^, MoPrP^*wild*^ and MoPrP^*mut*^ neuronal cells. Neuronal cells expressing PrP^C^, regardless of their type, were shown to have higher susceptibility to MuLV infection in illumination microscopy. Neuronal cells expressing MoPrP^*wild*^, ZW, or PrP^C^ with the 3F4 epitope, or MoPrP^*mut*^ with the octarepeat deletion, PrPΔ, showed intense staining of both PrP and CAgag at a similar level. The location of PrP in these cells was primarily in cytosol and membrane before MuLV infection. After MuLV infection, PrP staining was observed in the nucleus, cytosol, and also membranes. MuLV infection was observed mainly in cytosol by detection of CAgag. Neuronal cells expressing P101L mutant type of PrP^C^, MoPrP^*mut*^, were also susceptible to MuLV infection. The PrP^C^ of P101L was mainly located in the nuclear portion of the cells, thus the overlapping between PrP^C^ and CAgag was not observed clearly through illumination microscopy. MuLV infections in astroglial cells were not affected by PrP^C^. Different from neuronal cells, astroglial cells were largely resistant to infection by MuLV. Green, PrP; Red, CAgag; Blue, DAPI; Yellow, Merge. Scale bar = 20 μm.

**Fig 4:**
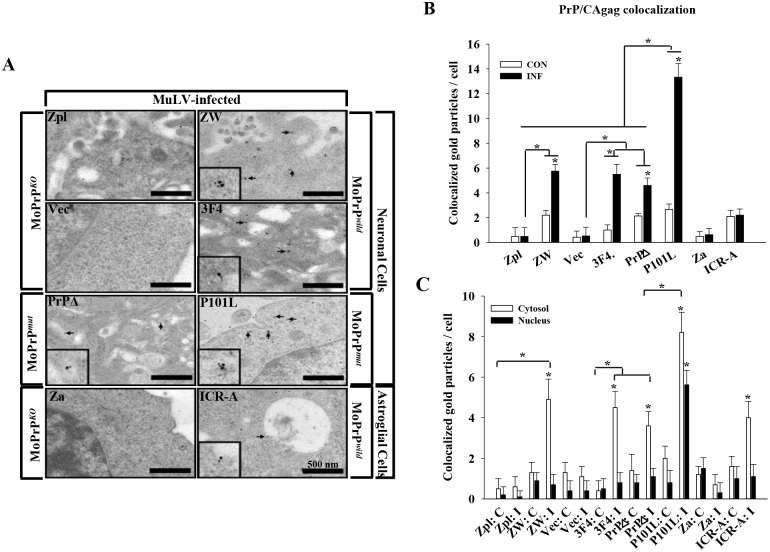
Colocalization of PrP^C^ and CAgag proteins in MuLV-infected cells. (A) Cells infected with MuLV were labeled with immunogold particles to observe colocalization of PrP^C^ and MuLV using electron microscopy. PrP^C^ and MuLV were conjugated with 10 nm-gold particle and 15 nm-gold particle, respectively. Particles due to colocalization were not observed in PrP^-/-^ cells, regardless of the cell type. In PrP^+/+^ neuronal cells, ZW, 3F4, PrPΔ, and P101L, PrP^C^-CAgag colocalization was easily observed, whereas colocalization was very rare in PrP^+/+^ astroglial cells, ICR-A. Black arrows indicate the colocaloization of PrP^C^ and CAgag. Scale bar = 500 nm. (B, C) Quantitation of the experiment shown in (A). Colocalization was assessed in whole sections (B) and in nuclear and cytosol fractions separately (C) by counting the paired immunogold particles. PrP^+/+^ neuronal cells showed markedly higher numbers of pairings of PrP^C^-CAgag gold particles compared to PrP^-/-^ cells and non-infected cells (*p < 0.001). Particles in the latter 2 groups were non-specific deposits. P101L PrP^+/+^ cells had significantly more PrP^C^-CAgag gold particles pairings than other PrP^+/+^ neuronal cells, ZW, 3F4, and PrPΔ (*p < 0.001). The small number of particles seen in PrP^-/-^ cells are background immunogold deposits.

In the normal cells, PrP^C^ is localized on the cell surface and in the cytosol of a subset of neurons in the hippocampus, neocortex, and thalamus of mouse brain [27]. PrP^C^-positive neuronal and astroglial cell lines expressed PrP^C^ on the cell surface and in the cytosol under normal conditions (Fig 3, S3 Fig). The one exception was the P101L-expressing neuronal cells, which expressed 80% of PrP protein in the nucleus, whereas only 20% was expressed in the cytosol (Fig 3, S3 Fig). This could be correlated with the fact that CAgag staining in P101L-expressing neuronal cells seems to be less compared to other type of PrP^C^-expressing neuronal cells. After MuLV infection, PrP^C^ expression levels were up-regulated in all PrP-positive neuronal cell lines, however, astroglial cell lines showed no difference between control and MuLV-infection conditions (Fig 3, S3 Fig). MuLV protein (CAgag) was detected in the cytosol portion after MuLV infection; PrP expression was observed in both the nucleus and the cytosol. PrP^+/+^ neuronal cells were stained much more extensively than PrP^-/-^ cell lines for CAgag after MuLV infection (Fig 3, S3 Fig). To verify the colocalization of PrP and MuLV which was observed in immunofluorescence results, immunogold labeling was assessed by electron microscopy using anti-3F10 PrP-detecting antibody and anti-CAgag-detecting antibody (Fig 4). PrP/CAgag colocalization was observed in PrP-expressing neuronal cell lines (Fig 4). Quantification of PrP/CAgag colocalization was done by counting the colocalized immunogold particles; the specificity of the reactions was established by 15 nm gold conjugated secondary antibody for CAgag, whereas for PrP, 10 nm gold particles were used. In agreement with immunofluorescence data, PrP/CAgag colocalized immunogold particles were significantly higher in PrP-expressing neuronal cells compared to CA/gag reactive particles seen in PrP^-/-^ cells (*p* < 0.001) (Fig 4). In PrP^-/-^ cells, there were 1~2 non-specific immunogold particles which are regarded as background. Results for astroglial cells did not reveal a difference in particles based on PrP presence. Colocalized particles were counted separately for the cytosol and nucleus, and in all PrP-expressing neuronal cells. There was significantly higher colocalization in the cytosol (*p* < 0.001)(Fig 4). These results are consistent with findings using immunofluorescence shown in Fig 3.

### Effect of MuLV infection on PrP^C^ expression and biochemical characteristics of PrP^C^ after MuLV infection

**Fig 5:**
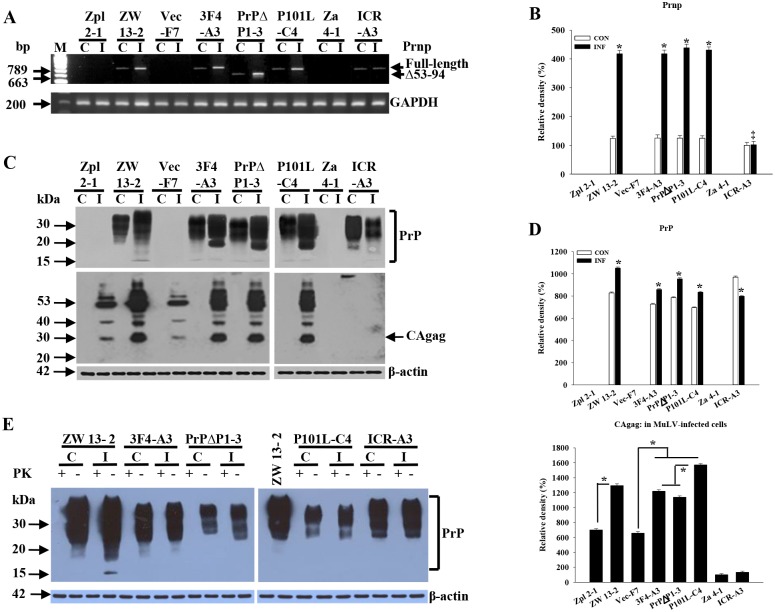
Correlation of PrP^C^ and MuLV infection in neuronal and astroglial cells. (A) *Prnp* expression levels in *Prnp*^+/+^ neuronal and glial cells. *Prnp*^+/+^ neuronal cells showed up-regulation after MuLV infection. *Prnp*^+/+^ astroglial cells showed no statistically significant differences from uninfected cells. (B) Densitometry analysis of *Prnp* expression showed significant differences between infected and control neuronal cells (*p < 0.05). There was no difference in expression of *Prnp*^+/+^ between control and infected ICR-A3. (C, D) In neuronal cells, significant correlation was observed between PrP^C^ expression and MuLV antigen. PrP^C^ protein levels were up-regulated in MuLV-infected neuronal cells, but expression was down-regulated in astroglial cells. PrP^+/+^ cells showed significantly higher levels of CAgag than PrP^-/-^ cells (*p < 0.01). Cells expressing P101L mutant form showed significantly higher levels of CAgag than wild-type and octarepeat deletion forms of PrP^C^ (*p < 0.05). Astroglial cells showed significantly lower levels of MuLV infection than either PrP^-/-^ or PrP^+/+^ neuronal cells (†*p* < 0.01). (E) The biochemical character of PrP^C^ in non-infected and MuLV-infected cells was investigated using PK treatment (20 μg/mL). Expressed PrP showed no PK-resistant form in either non-infected or MuLV-infected neuronal and astroglial cells.

In neuronal cells, MuLV infection affected the expression of PrP with regard to both mRNA and protein levels (*p* < 0.05) (Fig 5). After MuLV infection, PrP expression levels increased in neuronal cells, whereas astroglial cells showed decreased expression levels of PrP (Fig 5). The PrP produced in MuLV-infected cells was PK-sensitive (Fig 5). In neuronal cells, ZW, 3F4, PrPΔ and P101Ls, *prnp* expression levels of mRNA in MuLV-infected cells were increased by 3-fold compared to non-infected cells (*p* < 0.05). However, astroglial cells, ICR-A, showed no difference of PrP^C^ expression levels between MuLV-infected and non-infected cells (Fig 5). GAPDH was used as a housekeeping control. The protein expression levels of PrP^+/+^ neuronal cells were also up-regulated 1.3–1.6-fold in MuLV-infected compared to non-infected cells (*p* < 0.05). In contrast, astroglial cells showed depletion of PrP^C^ levels by 1.4-fold after MuLV-infection (*p* < 0.05) (Fig 5). Expression of PrP^C^ in neuronal cells showed agreement in both mRNA and protein. In astroglial cells, mRNA levels of PrP^C^ showed no difference between MuLV-infected and non-infected cells, but protein levels were down-regulated significantly. Infection levels of MuLV were estimated by Western blot of CAgag (Fig 5). PrP^+/+^ cells, ZW, 3F4, PrPΔ and P101Ls, showed 2–2.1-fold higher amounts of MuLV-infection than PrP^-/-^ cells, Zpl and Vec. However, no difference was observed between PrP^+/+^ astroglial cells, ICR-A, and PrP^-/-^ astroglial cells, Za. Also, both PrP^+/+^ and PrP^-/-^ astroglial cells showed a 35-fold lower infectivity than PrP^+/+^ neuronal cells and 7-fold lower infectivity compared to PrP^-/-^ neuronal cells (*p* < 0.01) (Fig 5). MuLV-infected and non-infected cells were treated with proteinase K (PK) to determine whether the PK-resistant scrapie form of PrP, PrP^Sc^, was generated (Fig 5). In both MuLV-infected and non-infected cells, no PK-resistant form was generated. β-actin was used as the housekeeping control protein. Results depicted in Fig 5 showed higher levels of CAgag in PrP-positive cells than in PrP-negative cells, confirming the immunocytochemical studies in Fig 3.

### PrP regulates galectin-1,-3,-6 proteins and affects MuLV infectivity but PrP with only galectin-3 and -6 involves in plaque formation

**Fig 6:**
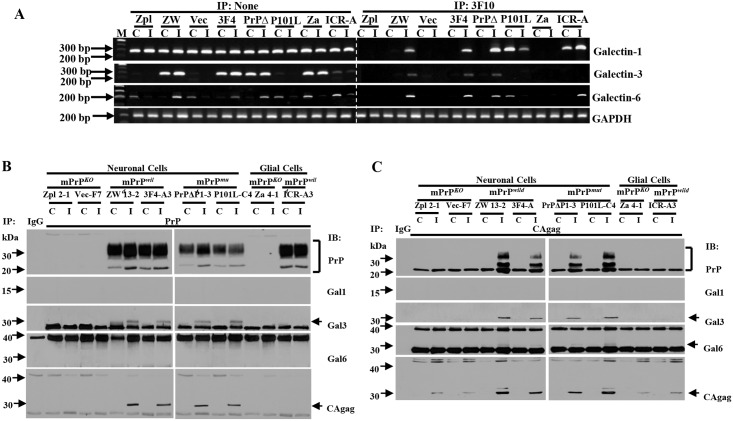
Expression and binding activity of PrP^C^ with galectin-1,-3, and -6 mRNAs and proteins. (A) Expression of mRNA levels of galectin-1, -3, and -6 was observed by quantitative RT-PCR method. Binding activity of PrP^C^ with galectin-1, -3, and -6 mRNAs was investigated by immunoprecipitation of mRNA-protein complex method using anti-PrP antibody (anti-3F10). Galectin-1 mRNA is constitutively expressed regardless of no infection or MuLV infection. Galectin-3 mRNA expression is closely related with PrP^C^ expression, thus it is detected in PrP^C^-expressing neuronal cells but not in PrP^-/-^ neuronal cells. Galectin-6 mRNA expression is related to MuLV infection in PrP^C^-expressing cells but not in PrP^-/-^ cells, P101L cells and PrP^+/+^ astroglial cells. Binding activity of PrP^C^ to galectin mRNAs was closely related to MuLV infection in PrP^+/+^ cells. For P101L cells, the binding activity to galectin-1 and -6 was unusual in that binding occurred in non-infected cells. This may have contributed to the large plaque size seen in P101L cells. (B) Anti-PrP (3F10) antibody was used to proceed immunoprecipitation then anti-PrP (3F10) (27–33 kDa), -galectin-1 (Gal1)(14 kDa), -galectin-3 (Gal3)(31 kDa), -galectin-6 (Gal6)(32 kDa), and –CAgag (30 kDa) antiboties were used to detect expression level of each protein. PrP was detected in all PrP expressing cell lines: ZW, 3F4, PrPΔ, P101L, and ICR cell lines regardless of infected or non-infected. CAgag and Gal3 was detected in MuLV-infected neuronal cell lines: ZW, 3F4, PrPΔ, and P101L cell lines. Gal1 and Gal6 was not detected in any of cell lines regardless of infected or non-infected. (C) Anti-CAgag antibody was used to proceed immunoprecipitation then anti-PrP (3F10) (27–33 kDa), -galectin-1 (Gal1)(14 kDa), -galectin-3 (Gal3)(31 kDa), -galectin-6 (Gal6)(32 kDa), and –CAgag (30 kDa) antiboties were used to detect expression level of each protein. CAgag was detected in all MuLV infected cells and Gal1 was not detected. PrP, Gal3, and Gal6 was detected at MuLV-infected PrP^+/+^ cells.

Galectin-1 and -3 are known to be involved in HIV infection [19], and galectin-6 was screened to be up-regulated in PrP^-/-^ neuronal cells by microarray. To find the connections between PrP and galectin-1, -3 and -6 proteins, we performed quantitative RT-PCR and immunoprecipitation RT-PCR (IP-PCR) using anti-3F10 PrP antibody, Western blot, and immunoprecipitation (IP) (Fig 6). In Fig 6A and S4A–S4C Fig, mRNA levels of galectin-1 showed no difference between non-infected and MuLV-infected cells. Galectin-3 mRNA was expressed in PrP^+/+^ neuronal cells but not in PrP^-/-^ cells. For galectin-6, mRNA was expressed to a greater extent in MuLV-infected vs. control PrP^+/+^ neuronal cells, whereas expression in controls was greater than infected in astroglia and in PrP^-/-^ cells. Interestingly, the mRNA specific for each of these galectins was present in immunoprecipitates produced by 3F10 anti-PrP antibody. The galectin proteins were also present in the 3F10 and CAgag immunoprecipitates (Fig 6). Infection with MuLV increased the levels of galectin-3 and -6 in PrP^+/+^ cells (S4 Fig). CAgag was also found in the 3F10 immunoprecipitates. In non-IP preparations, PrP protein-expression in astroglial cells was decreased by 50% in MuLV-infected cells compared to non-infected, whereas after IP, the PrP level found in precipitates was 1.5-fold higher in infected cells. The data in Fig 6B and S4 Fig shows that after MuLV infection of neuronal cells, CAgag levels were higher in PrP^+/+^ cells than in PrP^-/-^ cells and that MuLV infection increased the level of PrP^C^ detectable by Western blot. These results confirm the data shown in Figs 2 and 3. 3F10 immunoprecipitation showed PrP has binding activity only with galectin-3 among three galectin proteins we tested and also CAgag has detected (Fig 6). CAgag immunoprecipitation showed MuLV has binding activity with galectin-3 and -6 and also precipitates PrP together (Fig 6). Taken together, we conclude that PrP^C^, galectin-3 and -6 and CAgag proteins are interacting at the molecular level in MuLV-infected cells.

## Discussion

PrP^C^ is suggested to be a ligand or receptor for cellular proteins or metals [28]. This study was performed to determine if PrP^C^ has a role in exogenous virus infection. Previous work reported the possibility of interaction between PrP^C^ and endogenous MuLV, which increased MuLV replication and may have increased the rate of PrP^Sc^ production [29, 30]. These reports suggested the possibility that interaction between PrP^C^ and MuLV is involved in the progression of neurodegeneration. Ecotropic MuLV is known to induce virus-mediated cell fusion with certain cell lines such as the rat cell line XC and mouse cell line SC-1 [31]. We imported the cell fusion system used to assay MuLV and applied it to experimentally infected PrP^-/-^ and PrP^+/+^ hippocampal neuronal and glial cell lines. Using the same MuLV pool to infect all of the cell lines, we found that the plaque yield for PrP^-/-^ cell lines was approximately 3-fold less than for ZW, 3F4 and PrPΔ cell lines and 10-fold less than for P101L cells. The octarepeat deletion form of PrP is known to be involved in oxidative stress [32, 33], however, the octapeptide portion is not implicated in viral infection. Neither the absence of the octapeptide repeat nor the presence of the 3F4 epitope produced plaque numbers different from wild-type (ZW) cells. In contrast, the presence of the P101L mutant increased the plaque number compared to the other three PrP^+/+^ cell lines. The concept that PrP^C^ has an effect on MuLV is strengthened by the fact that one of the four cell lines (P101L) had higher numbers of plaques and increased plaque size compared to the other three PrP^+/+^ lines. This result indicates that there is a very specific interaction between PrP^C^ and MuLV that can be determined by different PrP^C^ sequences.

Although the aim of this study was to assess the role of PrP^C^ in the MuLV replication cycle, our results also suggest effects of MuLV on PrP^C^: following MuLV infection, there is an increase in PrP^C^ staining seen in both immunocytochemistry (Fig 3, S3 Fig) and in Western blot (Fig 5). The PK sensitivity of PrP^C^ was not affected by the presence of MuLV (Fig 5). This data indicates that PrP^C^ is a scavenger protein, which shows increased expression after recognition of MuLV, and that galectins 3/6 help PrP^C^ to interact with MuLV. In addition, MuLV plaque formation is correlated with PrP^C^ expressions and its susceptibility to aggregate/misfold.

It was surprising that despite the difference in plaque counts between PrP^-/-^ neuronal cell lines and three of the PrP^+/+^ lines, the size of plaques did not differ. Since galectins are a family of fifteen proteins in mammals [19] we postulate that other galectin family proteins which do not require PrP^C^ to form plaques can involve in determining size and forming plaques.

Further evidence suggesting an effect of MuLV on scrapie was obtained in the following findings: 1) Among three mouse strains, scrapie incubation periods were shortest in the strain with the highest MuLV titer and longest in the strain with virtually no MuLV in brain [34]; 2) The level of immunostaining for PrP^Sc^ was higher in the brains of mice with high levels of MuLV compared to a strain with little or no brain MuLV [30]; and 3) In sheep scrapie, the level of scrapie agent replication increased 2-fold if persistently infected microglia cells were coinfected with a ruminant derived retrovirus [35]. In summary, viral infection induces PrP^C^ expression that serves as a substrate for faster PrP^Sc^ amplification.

Since galectins are known to be involved in cell-cell fusion after viral infection, we estimated that the viral receptor effect of PrP is coupled with galectin proteins. Galectin proteins are known to recognize various microorganisms by protein-carbohydrate recognition and now appear to be involved in some neuronal recognition processes [19]. Galectin-1 is known for its anti-inflammatory activities and acts by blocking or attenuating signaling events. Recently, it has been shown to be a receptor for HIV-1. Galectin-3 has pro-inflammatory activity and has been proposed as a ‘danger signal’ among the intracellular galectins [23]. The function of galectin-6 is not known. We postulate that galectin-6 functions as a ‘cell-cell adhesion’ molecule and may participate in endocytosis of virus into cells. We conclude that PrP^C^ is involved in regulating the galectin proteins when neuronal cells are invaded by viruses. Evidence for this is the presence of galectin-3 and -6 in PrP^+/+^ but not in PrP^-/-^ and an increase in galectin-3 and -6 in immunoprecipitates resulting from anti-PrP antibody (Fig 6). It is interesting that these precipitates also contained CAgag in MuLV-infected neuronal cells. Furthermore, immunoprecipitates from a number of PrP^+/+^ lines (ZW, 3F4, PrPΔ) also contained mRNA of galectin-1, -3 and -6. Galectin-1 was constantly detected in both infected and non-infected cells and had no reaction in immunoprecipitations which suggests and confirms galectin-1 function as an anti-inflammatory protein. The incorporation of these molecules in the immunoprecipitates reflect an association that is involved in the replication of MuLV and/or PrP.

From these results, we can postulate that there may be another type of inherited mutant PrP^C^ which is present in fatal familial insomnia (FFI), another human prion disease. It would be interesting to determine if a cell line from FFI would yield results similar to or different from the P101L cell line.

PrP^-/-^ cells were infected by MuLV as evidenced by the CAgag found after exposure to the virus. If one assumes that PrP^C^ acts as a receptor for MuLV, then it follows that an alternative receptor exists. In astroglial cells, the presence of PrP^C^ did not affect MuLV infection. Astroglial cells were not infected with MuLV with or without PrP^C^. For astroglial cells MuLV could be toxic rather than infectious since MuLV-infected astroglial cells could not form the plaques and most cell were dead (Fig 2, S2 Fig).

The findings in this study explains that PrP^C^ functions as a receptor that augments MuLV uptake and thereby increases the number of plaques. The larger plaques seen in the P101L mutant line would be based on a more rapid spread of virus from the original infectious centers to neighboring cells producing enlarged plaques. Another explanation for the results presented is the potential effect of the interaction of PrP^C^ with the replication of MuLV. A series of studies [25, 36–41] have found that PrP can function, much as the NCp7 protein in HIV-1 replication, in aiding RNA dimerization and pro-viral DNA synthesis by reverse transcriptase. Human PrP can also accelerate DNA binding and strand transfer in the HIV replication cycle. It is certainly possible that mouse PrP could interact with the MuLV synthesis mechanism to affect virus yield. In relation to the difference in plaque numbers between PrP^+/+^ and PrP^-/-^, a positive effect on virus replication would lead to more rapid spread of virus, thereby affecting sufficient cells to yield visible plaques. The larger plaques seen in P101L would be related to greater enhancement of replication by the mutant sequence. In GSS cases, P102L *PRNP* mutation with M129V and A117V *PRNP* mutation are reported to enhance plaque numbers in the brain [42, 43]. Plaques and PrP correlation is so far only prion diseases issue and no reports related with other viral diseases so far.

**Fig 7:**
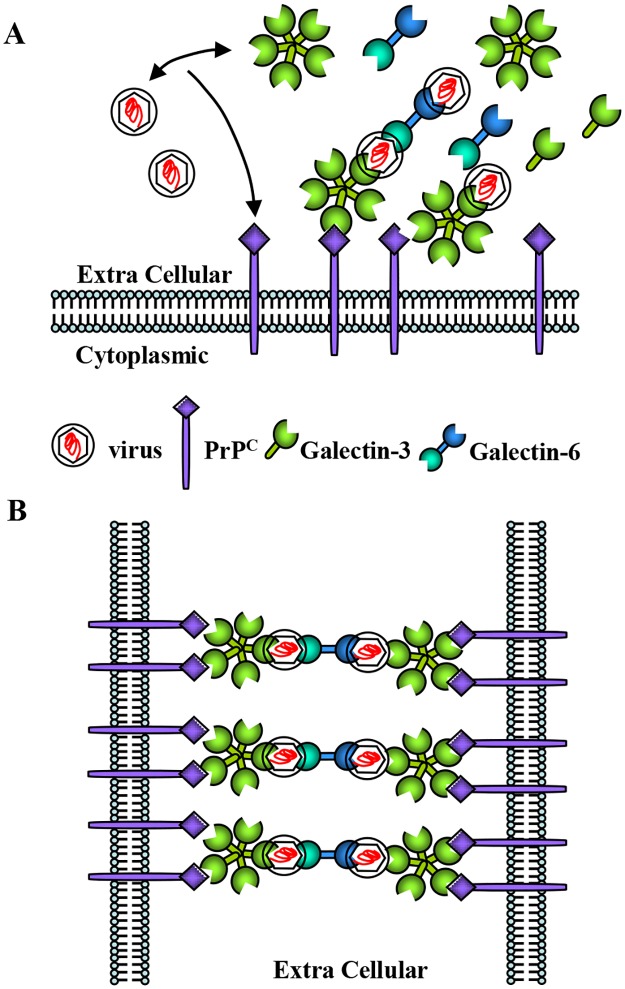
The cell-cell adhesion mechanisms to form plaques. (A) Scheme of PrP-MuLV-Gal interaction in cell membrane. (B) Based on our data between the cells PrP-Gal3 has binding activity and Gal3-CAgag-Gal6 shows binding activity. To produce the plaques, Gal3 is suggested to combine between PrP and MuLV. Gal6 is suggested to combine two different viruses to form plaques.

We postulate that in MuLV infection, PrP^C^-galectin binding activity is affecting viral infectivity in neuronal cells. Our results suggest that PrP^C^ in conjunction with galectin-3 and/or galectin-6 acts as a major receptor in MuLV infection (Fig 7). Galectin-3 is postulated to mainly capture the virus particles and bind with PrP^C^s. Galectin-6 may, in part, be involved in capturing the virus particles, but mainly is thought to participate in cell-cell adhesion mechanism. There is no evidence that galectin-1 interacts with PrP^C^, also it shows no changes of expression level even if cells are infected with MuLV. These data suggest that galectin-1 may participate in regulation of antiviral immunity and homeostasis. Note that the relevance of the subtle differences in galectin specificity and affinity to PrP^C^ which determine the different recognition effects. The intriguing difference of plaque forming mechanism between P101L and other PrP^+/+^ cell lines remain as a further study. The details of PrP^C^ and galectin proteins binding activity and the mechanism that leads to higher MuLV susceptibility in PrP^+/+^ compared to PrP^-/-^ cells should also be examined further.

## Materials and Methods

### Ethics statement

All animal experimentation has been conducted in accordance with the guidelines laid down by the NIH (NIH guide for the Care and Use of Laboratory Animals) in USA and by the Hallym Medical Center for the Institutional Animal Care and Use Committee. All use of mice was approved by the Hallym Medical Center for the Institutional Animal Care and Use Committee (IACUC, protocol# HMC 2011-0-0115-08). Briefly, animals were euthanized using carbon dioxide and brains were removed and placed in cell culture media.

### Cell culture, transfection and generation of stable cell lines

PrP^C^-deficient cell lines and wild-type cell lines were cultured under normal conditions [24]. Zpl cells expressing transfected PrP^C^ were generated by transfection with a 3F4-pcDNA3.1/zeo+ (PrP wild-type), PrP(Δ53–94)-pcDNA3.1/zeo+ (octarepeat deletion form of PrP) and a P101L-pcDNA3.1/zeo+ (GSS mutant PrP type)[44, 45]. Empty vector, pcDNA3.1/zeo+ (Invitrogen), was used as an additional negative control line. Nucleotide sequences of the cloned PrP genes were confirmed by an ABI377 automatic sequencer using a *Taq* dideoxy terminator cycle sequencing kit (ABI, Foster City, CA, USA). Transfected cells were selected by culturing the cells in DMEM containing 250 μg/mL zeocin (Invitrogen) for 4 weeks. PrP-expressing cells were screened by RT-PCR using primers detecting *Prnp* (Table 1) and Western blot using anti-3F4 at 1:10,000 (mouse monoclonal, supplied by Dr. R. Kascsak of the New York State Institute for Basic Research, Staten Island, NY, USA) and anti-3F10 at 1:1000 (mouse monoclonal)[46]. Expression levels of protein were quantified by densitometer (GS-800, Bio-Rad, CA, USA).

### Establishment of *Prnp*^-/-^ astroglia cell lines and characterization

Primary astroglia cells were cultured from 3-day neonates from Zür I mice (contributed by Dr. A. Aguzzi of Zürich University, Switzerland)[47]. All use of mice was approved by the Hallym Medical Center for the Institutional Animal Care and Use Committee (IACUC, protocol# HMC 2011-0-0115-08). Briefly, animals were euthanized and brains were removed and placed in cell culture media.

Cells were cultured with culture media (DMEM with 10% FBS, 100 unit/mL penicillin and 100 μg/mL streptomycin, Gibco BRL), incubated at 37°C in 5% CO_2_ and transfected with SV40 large T antigen containing vector (φ SV40, provided by Dr T. Onodera, Tokyo University) using 8 μg/mL of hexadimethrine bromide (Sigma-Aldrich, Cat# H9268)[26]. After immortalized cells were selected, immortalization was confirmed by Western blot using anti-SV40 at 1:1000 (mouse monoclonal, EMD Millipore, Cat# MABF121), and PrP expression was confirmed using anti-3F10. β-actin at 1:10,000 (mouse monoclonal, Sigma, Cat# A5441) was used as a housekeeping control protein. Morphological observation was estimated by inverted microscopy (Olympus, USA). The cell proliferation was estimated by cell counting [25]. Cell type was confirmed by Western blot and immunocytochemistry. For Western blot, anti-GFAP was used at 1:5000 (rabbit poly, DAKO, Cat# Z0334) and anti-MAP2 at 1:1000 (mouse monoclonal, EMD Millipore, Cat# MAB3418). For immunocytochemistry, anti-GFAP was used at 1:100 (rabbit poly, DAKO), anti-MAP2 at 1:50 (mouse monoclonal, EMD Millipore), and anti-CNPase at 1:50 (mouse monoclonal, Sigma-Aldrich, Cat# C5922). A horseradish peroxidase (HRP)-conjugated secondary antibody (Pierce) was used for Western blot and a fluorescein isothiocynate (FITC)-conjugated or a Tetramethyl Rhodamine IsoThioCyanate (TRITC)-conjugated secondary antibody was used for immunocytochemistry (Zymed). 10 μM of 4',6-Diamidino-2-phenyindole, dilactate (DAPI, Sigma-Aldrich, Cat# D9564) staining was applied as a cellular marker, then preparations were observed using confocal microscopy (Zeiss, Germany).

#### Reverse **transcriptase polymerase** chain reaction (RT-PCR) analysis

Total RNA was extracted from mouse brains using Trizol reagent (BRL) according to the manufacturer’s protocol, and then cDNA was synthesized from 2 μg of total RNA using AMV reverse transcriptase (Promega) and oligo (dT) primer. The mixture was incubated at 25°C for 10 min, 42°C for 1 hr, and the reaction heat-inactivated at 95°C for 5 min and kept at 4°C until use. PCR cycling parameters were: 94°C, 3 min; 72°C 1 min. Primers and conditions are shown in Table 1. Nucleotide sequences of the PCR products were confirmed by an ABI377 automatic sequencer using a *Taq* dideoxy terminator cycle sequencing kit (ABI, Foster City, CA, USA).

### XC/UV plaque assay for ecotropic MuLV

In order to quantitate the MuLV infection, 12-month-old SAMP8 brains were homogenized at 10% w/v in DMEM by 20 strokes in ground glass tissue homogenizers. Ecotropic MuLV which is capable of infecting only mouse cells was quantitated using the XC/UV plaque assay as previously described [48, 49]. Previously in our work, titer of MuLV from SAMP8 mice was tested by serial dilution and multiplicity of infection (MOI) was tested. Briefly, PrP^-/-^ and PrP^+/+^ cells were plated onto 60 mm dishes (10^5^ cells/dish) in normal culture media (DMEM10A). The following day, medium was replaced with 3 mL/dish DMEM10A with 25 μg/mL DEAE-dextran (Sigma-Aldrich). Dilutions of homogenized SAMP8 brain tissues were added to dishes in 1 mL volumes. The following day, medium was changed to DMEM10A (4 mL/dish). Dishes were incubated for 4 days, then the medium was aspirated and exposed to 30 s UV irradiation. Immediately after UV exposure, 3.0 x 10^5^ XC cells in 4 mL DMEM10A were added to each dish. After 24 h incubation, the medium was discarded. Cultures were washed once with phosphate-buffered saline (PBS), fixed with 100% methanol for 5 min and stained with hematoxylin (Fisher Scientific, USA) for 5 min. Hematoxylin was discarded, the cultures washed twice with tap water, and the plaques counted under a dissecting microscope at 40x magnification. Plaque counts at various dilutions were corrected to the 1% dilution of tissue (yielding at titer per 10 mg tissue) by multiplying the plaque count by the dilution factor [48].

### Measurement of plaque size of the cells

The plaque size of the cells was observed under anatomic microscope (Nikon, Japan) and measured by an objective micrometer (Shibuya, Japan).

### Detection of PrP and MuLV after infection of cells

PrP and MuLV infection were detected by RT-PCR and Western blot analysis. Prnp expression alteration after MuLV infection was detected by RT-PCR (Table 2). As a housekeeping control, GAPDH was used. Western blot analysis was performed using anti-3F10 (1:1000) to detect PrP^C^ and anti-CAgag (1:5000, goat polyclonal, Quality Biotech Inc.) to detect alteration of expression levels after MuLV infection. Proteinase K (PK, 20 μg/mL) was used at 37°C for 15 min to investigate the biochemical characteristic of PrP after MuLV infection.

**Table 2:** Primer sequences and conditions for RT-PCR.

Gene	Primer sequence 5′→3′	Annealing (°C) Cycles	Product size (bp)
SV40LT-Ag	(+): TGAGGCTACTGCTGACTCT	51	105
	(-): GCATGACTCAAAAAACTTAGCAATTCTG	40	
Prnp	(+): ATGGCGAACCTTGGCTACTGG	57	789
	(-): CCCTCATCCCACGATCAGGAAGATG	30	
Galectin-1	(+): TCAATCATGGCCTGTGGTCT	58	284
	(-): GCACACCTCTGTGATGCTCC	35	
Galectin-3	(+): AGGGAATGATGTTGCCTTCC	58	268
	(-): GATCCCCAGTTGGCTGATTT	35	
Galectin-6	(+): CAAGCACTTCGAGCTGGTGT	58	223
	(-): ATTGAAGACTGGGGGTCCTG	35	
GAPDH	(+): TGGTATCGTGGAAGGACTCATGAC	58	200
	(-): ATGCCAGTGAGCTTCCCGTTCAGC	35	

(+) Sense;

(-) Anti-sense

### Immunocytological methods

Cells were fixed with 4% paraformaldehyde in PBS, permeabilized with 0.2% Triton X-100 (Sigma-Aldrich) at room temperature (R.T.) for 10 min, treated with 5% normal donkey serum (Jackson, USA) in PBS at R.T. for 1 h, and washed with PBS. Cells were incubated with anti-3F10 (1:100, anti-PrP monoclonal) and anti-CAgag (1:100, anti-MuLV goat polyclonal, Quality Biotech Inc.). After secondary antibodies were conjugated, 10 μM DAPI (Sigma-Aldrich) was added, and cells were incubated at 37°C for 1 min and observed with confocal microscopy (Zeiss). DAPI staining was used as a cellular marker [26].

### Electron microscopy

Cells were fixed in 1.66% glutaraldehyde, 1.6% paraformaldehyde buffered with 0.1 M phosphate buffer on ice for 2 h as previously described [24]. Ultra thin sections (90 nm) were cut in a RMC MTXL ultramicrotome (Tucson, AZ, USA). Collected sections were immersed in a retrieval solution (pH 9, DAKO, Glostrup, Denmark) for etching then incubated for 15 min at 90°C [50]. Sections were blocked with 0.5% BSA in PBS for 20 min then incubated with anti-CAgag (1:100) in 0.5% BSA in PBS with 0.5 M NaCl for 60 min at 60°C. After washing with washing buffer [0.5% BSA in PBS including 0.5 M NaCl, 0.1% gelatin and 0.05% Tween-20], sections were incubated with an anti-goat IgG conjugated to 15 nm gold particles (Electron Microscopy Sciences, Hatfield, PA, USA) which was diluted at 1:50 in 0.5% BSA including 0.5 M NaCl for 60 min at 60°C. For double labeling, sections were incubated with anti-PrP antibody (3F4 or 3F10) (1:100) for 60 min at 60°C, followed by incubation with an anti-mouse IgG conjugated to 10 nm gold particles (Electron Microscopy Sciences, Hatfield, PA, USA) which was diluted at 1:50 for 60 min at 60°C. After washing with buffer, cells were postfixed with 2.5% glutaraldehyde in PBS for 10 min at R.T. Sections were counterstained with uranyl acetate and lead citrate. The sections were observed using a transmission electron microscope (JEM-1011, JEOL, Japan). In order to assess relation between CAgag and PrP, we counted the colocalized gold particles (15 nm for CAgag, 10 nm for PrP) within the cells from each cell line.

### Immunoprecipitation of mRNA-PrP^C^ complexes

To elucidate the mRNA regulation of PrP^C^, immunoprecipitation of mRNA-protein complex assaying method was used. Anti-3F10 (anti-PrP monoclonal) antibody was used to precipitate the binding mRNAs. All reagents and procedures were done according to a previous report [40]. After extraction of the PrP^C^-mRNA complexes, reverse transcriptase polymerase chain reaction (RT-PCR) was done in the usual manner (Table 2).

### Immunoprecipitation of protein-protein complexes

The cells were homogenized in 10% w/v into RIPA buffer [50 mM Tris-HCl (pH 7.4), 0.25% Na-oxycholate, 1 mM EDTA, 1 mM NaF, 1% NP-40, 150 mM NaCl, 1 mM Na_3_VO_4_, proteinase inhibitor cocktail] using glass homogenizers (Corning) then incubated on ice for 10 min. The homogenate was centrifuged at 15,000 x g for 20 min at 4°C, and the supernatants were collected. The supernatants were pre-cleared by incubating with protein A+G-agarose beads (Calbiochem) for 2 h at 4°C. After centrifugation, the supernatants (1 mg) were incubated with anti-3F10 antibody (anti-PrP monoclonal) or normal mouse IgG (Santa Cruz) as a negative control (2 μg) for 12 h at 4°C. After beads were added, samples were incubated for 2 h at 4°C. After washing, the bound materials were eluted by mixing with 2x sample buffer then boiled for 10 min. The eluted proteins were separated on 12–15% SDS-PAGE gels and immunoblotted with the following antibodies: anti-galectin-1 (1:1000, goat polyclonal, Santa Cruz, Cat# sc-19277), anti-galectin-3 (1:1000, rabbit polyclonal, Santa Cruz, Cat# sc-20157), anti-galectin-6 (1:1000, goat polyclonal, Santa Cruz, Cat# sc-31800), anti-CAgag (1:5000, goat polyclonal, Quality Biotech Inc), anti-PrP (1:1000, mouse monoclonal 3F10), and anti-β-actin (1:10,000, mouse monoclonal, Sigma-Aldrich, Cat# A5441).

### Statistical analysis

Quantitative analyses were performed by one-way ANOVA test. All data were reported as means ± SEM. Differences were considered significant at *P* value of less than 0.05, 0.01, and 0.001.

## Supporting Information

S1 Fig Cell-type characterization of *Prnp*^-/-^ and *Prnp*^*+/+*^ neuronal and astroglial cell lines from Zür I mice and wild-type mice. (A) Confirmation of deficient, full-length *Prnp*, 789 bp, and truncated *Prnp*, 663 bp, expression level in cell lines. (B) Analysis for the expression of PrP^C^ using anti-3F10 antibody in neuronal cell lines. (C) Confirmation of SV40LT-Ag expression (93 kDa) in Za and ICR-A cell lines. φSV40 cell lysate was used as a positive control. ICR and Zür I mouse brains were used as negative controls. (D) Western blot analysis for the expression of PrP^C^ using anti-3F10 antibody. (E) Characterization of cell types by Western blot analysis. Established cell lines were positive for anti-GFAP antibody, an astroglial marker, and negative for anti-MAP2, a neuronal cell marker. (F) Three types of cell marker antibodies were tested by immunocytochemistry to determine cell type. DAPI staining (blue) was used as a cellular marker. C6, positive control for astroglial cells; Zpl 2–1, a positive control for neuronal cells; HGB, a positive control for oligodendroglial cells. Scale bar = 40 μm.
(TIF)

S2 Fig Association of PrP^C^ and morphological appearance of plaques in different clones of PrP^-/-^ and PrP^+/+^ neuronal and astroglial cells. The XC/UV plaque assay was applied to 2 clones of each of the cell lines listed in Table 1 which were then compared for plaque morphology and for size (S1 Table). (A) Plaque size and morphology results for the 2 additional clones/cell line were similar to the same cell line for each clone shown in Fig 2A. (B) Two clones of MoPrP^*KO*^ or MoPrP^*wild*^ astroglial cells did not form plaques, results similar to the clones shown in Fig 2B. Scale bar = 0.1 mm.
(TIF)

S3 Fig Different susceptibility to MuLV infection of PrP^-/-^ and PrP^+/+^ neuronal cells. Two additional clones for each cell line were assessed for MuLV infectivity. Neuronal cells expressing PrP^C^, regardless of their type, were shown to have higher susceptibility to MuLV infection in illumination microscopy. (A) Similar to results in Fig 3, immunocytochemistry of neuronal cells expressing wild-type PrP^C^, ZW, or PrP^C^ with the 3F4 epitope or PrP^C^ with the octarepeat deletion, PrPΔ, showed intensive staining of both PrP and CAgag. The location of PrP and CAgag was similar to that seen in Fig 3. Before MuLV infection, PrP was observed in cytosol and membrane, whereas after MuLV infection, PrP staining was observed in nuclear, cytosol, and membrane structures. MuLV infection was observed mainly in cytosol by detection of CAgag. Neuronal cells expressing P101L mutant type of PrP^C^ were also susceptible to MuLV infection. The PrP^C^ of P101L was mainly located in the nuclear portion of the cells, thus the overlapping between PrP^C^ and CAgag was not observed clearly through illumination microscopy. (B) MuLV infections in astroglial cells were not affected by PrP^C^. Unlike neuronal cells, astroglial cells were largely resistant to infection by MuLV. Green, PrP; Red, CAgag; Blue, DAPI; Yellow, Merge. Scale bar = 20 μm.
(TIF)

S4 Fig Quantification of expression and binding activity of PrP^C^ with galectin-1, -3, and -6 mRNAs and proteins. (A-C) Quantitative expression of mRNA levels of galectin-1, -3, and -6 were observed by regular RT-PCR method. Binding activity of PrP^C^ with galectin-1, -3, and -6 mRNAs was investigated by immunoprecipitation of mRNA-protein complex method using anti-PrP antibody (anti-3F10). Increase in MuLV-infected compared to non-infected; *p < 0.01. Increase in non-infected compared to MuLV-infected; **p < 0.01. (D) Quantitative expression of protein levels of CAgag were observed by Western blot analysis. Protein-protein binding activity between PrP^C^ and CAgag were assayed by IP method using PrP antibody (anti-3F10). Difference in expression in MuLV-infected compared to non-infected cells; *p < 0.05. Increase in non-infected compared to MuLV-infected; **p < 0.01. Difference in expression in astroglial cells vs. neuronal cells; †*p* < 0.01. (E) Expression of protein levels of galectin-1, -3, -6, and CAgag was observed by Western blot analysis. Protein-protein binding activity between PrP^C^ and galectin-1, -3, -6, and CAgag was determined by IP method. Galectin-1 protein expression was constitutive in both non- and MuLV-infected cells as was seen for mRNA expression. Galectin-3 and -6 required PrP for expression at the protein level. CAgag, the MuLV protein, was detected in all MuLV-infected neuronal cells but at different levels between PrP^-/-^ and PrP^+/+^ cells. Binding activity (detected by 3F10 antibody) of PrP^C^ to galectin-1, -6, and to CAgag was closely related to PrP^+/+^ and to MuLV infection. Binding of those proteins from astroglial cells did not occur. Galectin-3 did not bind to PrP^C^, regardless of the cell type.
(TIF)

S1 Table MuLV plaque number assay in PrP^-/-^ and PrP^+/+^ neuronal and astroglial cell lines. (DOCX)

S2 Table Plaque size of PrP^-/-^ and PrP^+/+^ neuronal and astroglial cell lines. (DOCX)

## Abbreviations

CAgag: MuLV gag protein
MoPrP^*mut*^: mouse mutant PrP^C^
MuLV: murine leukemia endogenous retrovirus
PrP^C^: cellular prion protein
PrP^-/-^ and MoPrP^*KO*^: mouse PrP^C^-deficient
PrP^+/+^ and MoPrP^*wild*^: mouse wild-type PrP^C^ present
