# Supplementary figures and images for: Cellular Prion Protein Combined with Galectin-3 and -6 Affects the Infectivity Titer of an Endogenous Retrovirus Assayed in Hippocampal Neuronal Cells
Source: PLoS One. 2016 Dec 9;11(12):e0167293. doi: 10.1371/journal.pone.0167293 (PMC5147886; doi:10.1371/journal.pone.0167293)

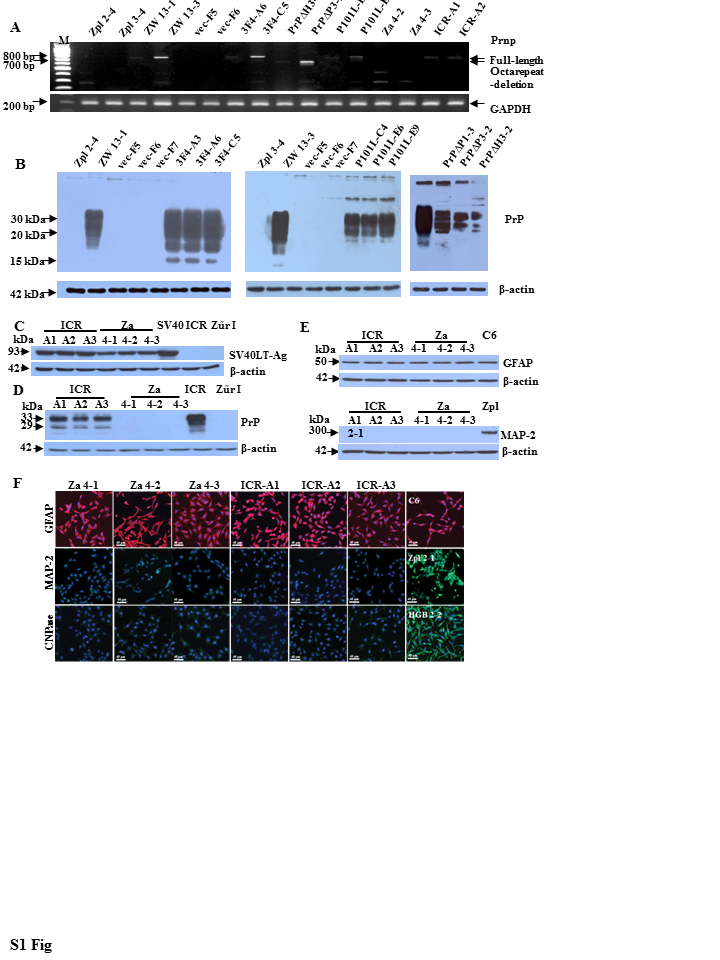

Supplement: S1 Fig — (A) Confirmation of deficient, full-length Prnp, 789 bp, and truncated Prnp, 663 bp, expression level in cell lines. (B) Analysis for the expression of PrPC using anti-3F10 antibody in neuronal cell lines. (C) Confirmation of SV40LT-Ag expression (93 kDa) in Za and ICR-A cell lines. φSV40 cell lysate was used as a positive control. ICR and Zür I mouse brains were used as negative controls. (D) Western blot analysis for the expression of PrPC using anti-3F10 antibody. (E) Characterization of cell types by Western blot analysis. Established cell lines were positive for anti-GFAP antibody, an astroglial marker, and negative for anti-MAP2, a neuronal cell marker. (F) Three types of cell marker antibodies were tested by immunocytochemistry to determine cell type. DAPI staining (blue) was used as a cellular marker. C6, positive control for astroglial cells; Zpl 2–1, a positive control for neuronal cells; HGB, a positive control for oligodendroglial cells. Scale bar = 40 μm. (TIF) [file pone.0167293.s001.TIF]

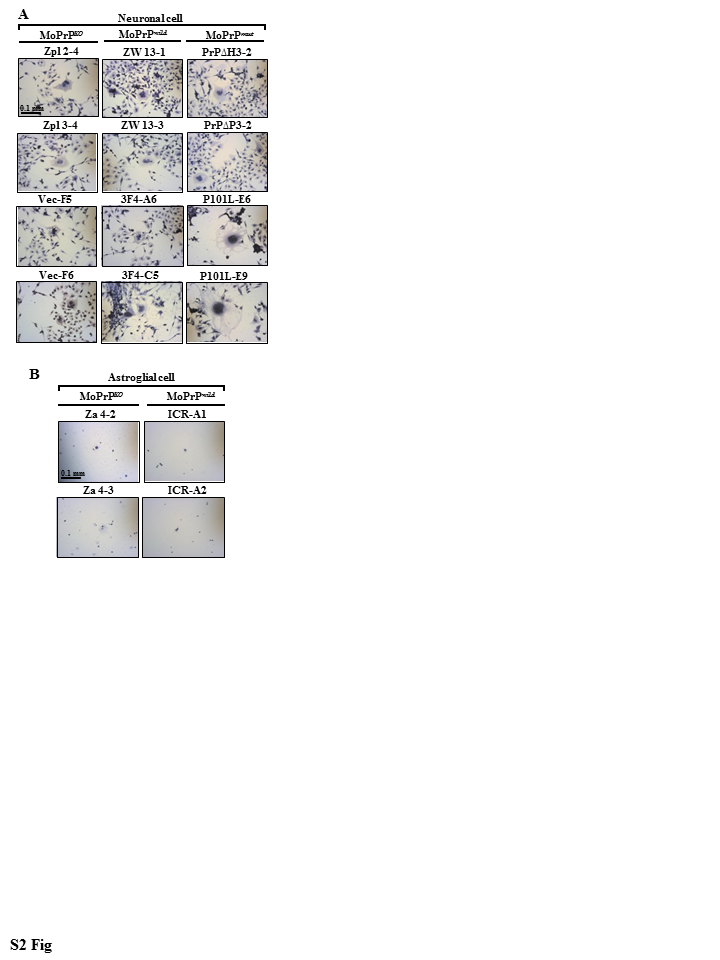

Supplement: S2 Fig — The XC/UV plaque assay was applied to 2 clones of each of the cell lines listed in Table 1 which were then compared for plaque morphology and for size (S1 Table). (A) Plaque size and morphology results for the 2 additional clones/cell line were similar to the same cell line for each clone shown in Fig 2A. (B) Two clones of MoPrPKO or MoPrPwild astroglial cells did not form plaques, results similar to the clones shown in Fig 2B. Scale bar = 0.1 mm. (TIF) [file pone.0167293.s002.TIF]

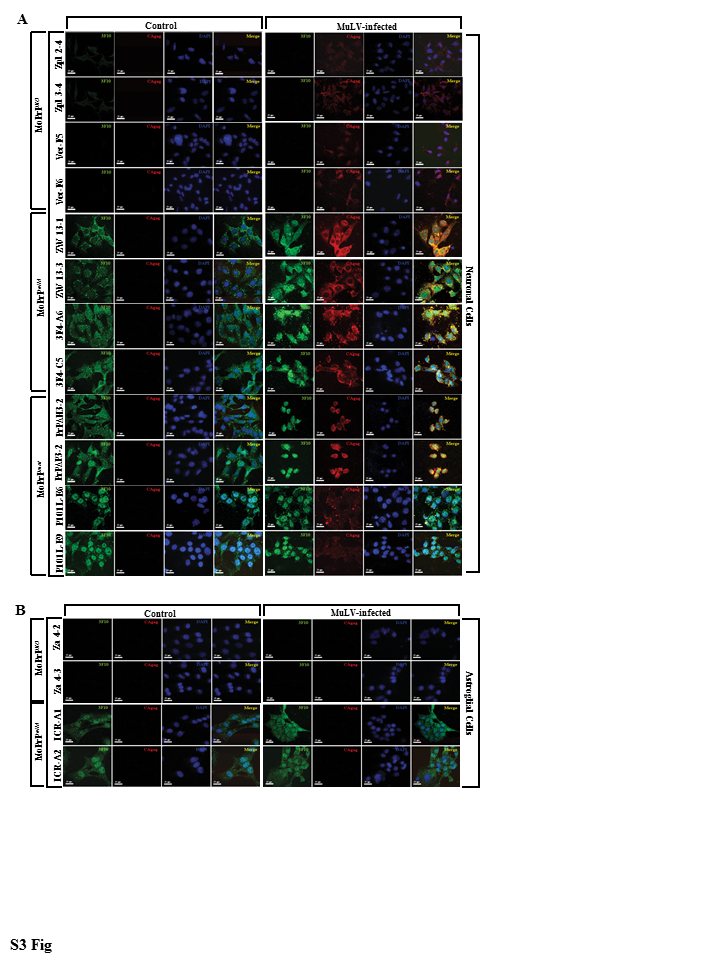

Supplement: S3 Fig — Two additional clones for each cell line were assessed for MuLV infectivity. Neuronal cells expressing PrPC, regardless of their type, were shown to have higher susceptibility to MuLV infection in illumination microscopy. (A) Similar to results in Fig 3, immunocytochemistry of neuronal cells expressing wild-type PrPC, ZW, or PrPC with the 3F4 epitope or PrPC with the octarepeat deletion, PrPΔ, showed intensive staining of both PrP and CAgag. The location of PrP and CAgag was similar to that seen in Fig 3. Before MuLV infection, PrP was observed in cytosol and membrane, whereas after MuLV infection, PrP staining was observed in nuclear, cytosol, and membrane structures. MuLV infection was observed mainly in cytosol by detection of CAgag. Neuronal cells expressing P101L mutant type of PrPC were also susceptible to MuLV infection. The PrPC of P101L was mainly located in the nuclear portion of the cells, thus the overlapping between PrPC and CAgag was not observed clearly through illumination microscopy. (B) MuLV infections in astroglial cells were not affected by PrPC. Unlike neuronal cells, astroglial cells were largely resistant to infection by MuLV. Green, PrP; Red, CAgag; Blue, DAPI; Yellow, Merge. Scale bar = 20 μm. (TIF) [file pone.0167293.s003.TIF]

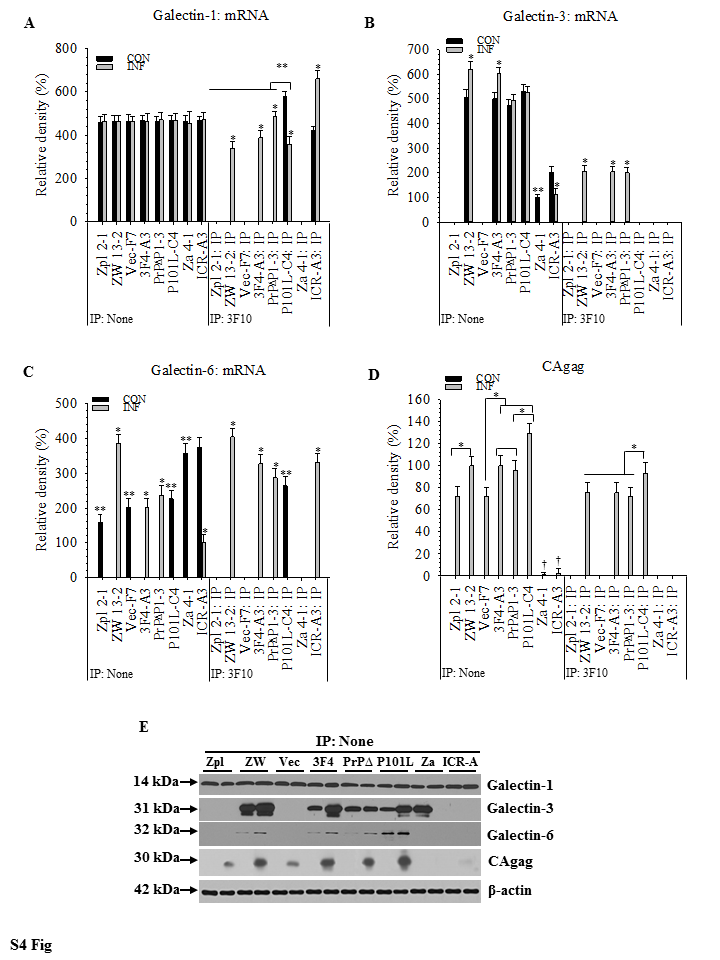

Supplement: S4 Fig — (A-C) Quantitative expression of mRNA levels of galectin-1, -3, and -6 were observed by regular RT-PCR method. Binding activity of PrPC with galectin-1, -3, and -6 mRNAs was investigated by immunoprecipitation of mRNA-protein complex method using anti-PrP antibody (anti-3F10). Increase in MuLV-infected compared to non-infected; *p < 0.01. Increase in non-infected compared to MuLV-infected; **p < 0.01. (D) Quantitative expression of protein levels of CAgag were observed by Western blot analysis. Protein-protein binding activity between PrPC and CAgag were assayed by IP method using PrP antibody (anti-3F10). Difference in expression in MuLV-infected compared to non-infected cells; *p < 0.05. Increase in non-infected compared to MuLV-infected; **p < 0.01. Difference in expression in astroglial cells vs. neuronal cells; †p < 0.01. (E) Expression of protein levels of galectin-1, -3, -6, and CAgag was observed by Western blot analysis. Protein-protein binding activity between PrPC and galectin-1, -3, -6, and CAgag was determined by IP method. Galectin-1 protein expression was constitutive in both non- and MuLV-infected cells as was seen for mRNA expression. Galectin-3 and -6 required PrP for expression at the protein level. CAgag, the MuLV protein, was detected in all MuLV-infected neuronal cells but at different levels between PrP-/- and PrP+/+ cells. Binding activity (detected by 3F10 antibody) of PrPC to galectin-1, -6, and to CAgag was closely related to PrP+/+ and to MuLV infection. Binding of those proteins from astroglial cells did not occur. Galectin-3 did not bind to PrPC, regardless of the cell type. (TIF) [file pone.0167293.s004.TIF]
