# Supplementary material for: Cellular Prion Protein Combined with Galectin-3 and -6 Affects the Infectivity Titer of an Endogenous Retrovirus Assayed in Hippocampal Neuronal Cells
Source: PLoS One. 2016 Dec 9;11(12):e0167293. doi: 10.1371/journal.pone.0167293 (PMC5147886; doi:10.1371/journal.pone.0167293)
Supplement: S1 Table — (DOCX) [file pone.0167293.s005.docx]

**S1 Table. MuLV plaque number assay in PrP^-/-^ and PrP*^+^*^/+^ neuronal and astroglial cell lines.**

| Cell line | No. of plaques | Average no. of plaques | Genotype | Cell type |
| --- | --- | --- | --- | --- |
| Zpl 2-1 | 42 ± 3.26 | 35.6 ± 5.68 | *Prnp*^-/-^ | Neuronal cell |
| Zpl 2-4 | 34 ± 4.55 |  |  |  |
| Zpl 3-4 | 31 ± 3.27 |  |  |  |
| ZW 13-1 | 136 ± 16.22 | 144.3 ± 28.43 | *Prnp^+^*^/+^ |  |
| ZW 13-2 | 121 ± 17.89 |  |  |  |
| ZW 13-3 | 176 ± 13.05 |  |  |  |
| Vec-F5 | 23 ± 0.92 | 24.6 ± 1.52 | *Prnp*^-/-^  Vector transfected |  |
| Vec-F6 | 26 ± 1.12 |  |  |  |
| Vec-F7 | 25 ± 1.11 |  |  |  |
| 3F4-A3 | 115 ± 5.01 | 112 ± 6.08 | *Prnp^+^*^/+^  Wild-type PrP |  |
| 3F4-A6 | 116 ± 3.66 |  |  |  |
| 3F4-C5 | 105 ± 7.05 |  |  |  |
| PrP∆H3-2 | 104 ± 2.22 | 107 ± 3.21 | *Prnp^+^*^/+^  Octarepeat region deleted PrP |  |
| PrP∆P1-3 | 110 ± 3.42 |  |  |  |
| PrP∆P3-2 | 109 ± 3.53 |  |  |  |
| P101L-C4 | 347 ± 11.29 | 363 ± 15.17 | *Prnp^+^*^/+^  P101L mutated PrP: human GSS type |  |
| P101L-E6 | 377 ± 12.98 |  |  |  |
| P101L-E9 | 366 ± 11.96 |  |  |  |
| Za 4-1 | 2 ± 1 | 1.5 ± 1 | *Prnp*^-/-^ | Astroglial cell |
| Za 4-2 | 1 ± 1 |  |  |  |
| Za 4-3 | 0 |  |  |  |
| ICR-A1 | 1 ± 1 | 1.5 ± 1 | *Prnp^+^*^/+^ |  |
| ICR-A2 | 0 |  |  |  |
| ICR-A3 | 2 ± 1 |  |  |  |

Ave., average of plaque number
