# Supplementary material for: Cellular Prion Protein Combined with Galectin-3 and -6 Affects the Infectivity Titer of an Endogenous Retrovirus Assayed in Hippocampal Neuronal Cells
Source: PLoS One. 2016 Dec 9;11(12):e0167293. doi: 10.1371/journal.pone.0167293 (PMC5147886; doi:10.1371/journal.pone.0167293)
Supplement: S2 Table — (DOCX) [file pone.0167293.s006.docx]

**S2 Table. Plaque size of PrP^-/-^ and PrP*^+^*^/+^ neuronal and astroglial cell lines.**

| Cell line | Plaque size Ave. (mm) | Ave.  (mm) | Genotype | Cell type |
| --- | --- | --- | --- | --- |
| Zpl 2-1 | 0.07 ± 0.015 | 0.07 ± 0.015 | *Prnp*^-/-^ | Neuronal cell |
| Zpl 2-4 | 0.07 ± 0.013 |  |  |  |
| Zpl 3-4 | 0.07 ± 0.015 |  |  |  |
| ZW 13-1 | 0.07 ± 0.011 | 0.07 ± 0.011 | *Prnp^+^*^/+^ |  |
| ZW 13-2 | 0.07 ± 0.012 |  |  |  |
| ZW 13-3 | 0.07 ± 0.011 |  |  |  |
| Vec-F5 | 0.065 ± 0.003 | 0.067 ± 0.002 | *Prnp*^-/-^  Vector transfected |  |
| Vec-F6 | 0.07 ± 0.002 |  |  |  |
| Vec-F7 | 0.066 ± 0.002 |  |  |  |
| 3F4-A3 | 0.068 ± 0.001 | 0.067 ± 0.001 | *Prnp^+^*^/+^  Wild-type PrP |  |
| 3F4-A6 | 0.067 ± 0.001 |  |  |  |
| 3F4-C5 | 0.066 ± 0.001 |  |  |  |
| PrP(∆53-94)H3-2 | 0.06 ± 0.003 | 0.063 ± 0.005 | *Prnp^+^*^/+^  Octarepeat region deleted PrP |  |
| PrP(∆53-94)P1-3 | 0.07 ± 0.005 |  |  |  |
| PrP(∆53-94)P3-2 | 0.06 ± 0.005 |  |  |  |
| P101L-C4 | 0.21 ± 0.076 | 0.27 ± 0.072 | *Prnp^+^*^/+^  P101L mutated PrP: human GSS type |  |
| P101L-E6 | 0.25 ± 0.066 |  |  |  |
| P101L-E9 | 0.35 ± 0.053 |  |  |  |
| Za 4-1 | 0.003 ± 0.0002 | 0.0027 ± 0.0005 | *Prnp*^-/-^ | Astroglial cell |
| Za 4-2 | 0.002 ± 0.0001 |  |  |  |
| Za 4-3 | 0.003 ± 0.0001 |  |  |  |
| ICR-A1 | 0.003 ± 0.0001 | 0.0027 ± 0.0001 | *Prnp^+^*^/+^ |  |
| ICR-A2 | 0.003 ± 0.0001 |  |  |  |
| ICR-A3 | 0.002 ± 0.0002 |  |  |  |

Ave., average of plaque number
